# Utilizing reflectance confocal microscopy for surveillance of neoplastic changes in penile lichen sclerosus: A report of two cases

**DOI:** 10.1016/j.jdcr.2024.05.028

**Published:** 2024-06-05

**Authors:** Hailey Konisky, Diana Mary Guevara Coteron, Betsy Chavez Morales, Albert Huho

**Affiliations:** a Albert Einstein College of Medicine, Bronx, New York; b Pontifical Catholic University of Argentina, Buenos Aires, Argentina; c Clinical and MOH’s Services, Upstate Dermatology, Castleton on Hudson, New York

**Keywords:** balanitis xerotica obliterans, genital lesions, lichen sclerosus, premalignant changes, reflectance confocal microscopy, squamous cell carcinoma

## Clinical presentation

### Case 1

**Fig 1:**
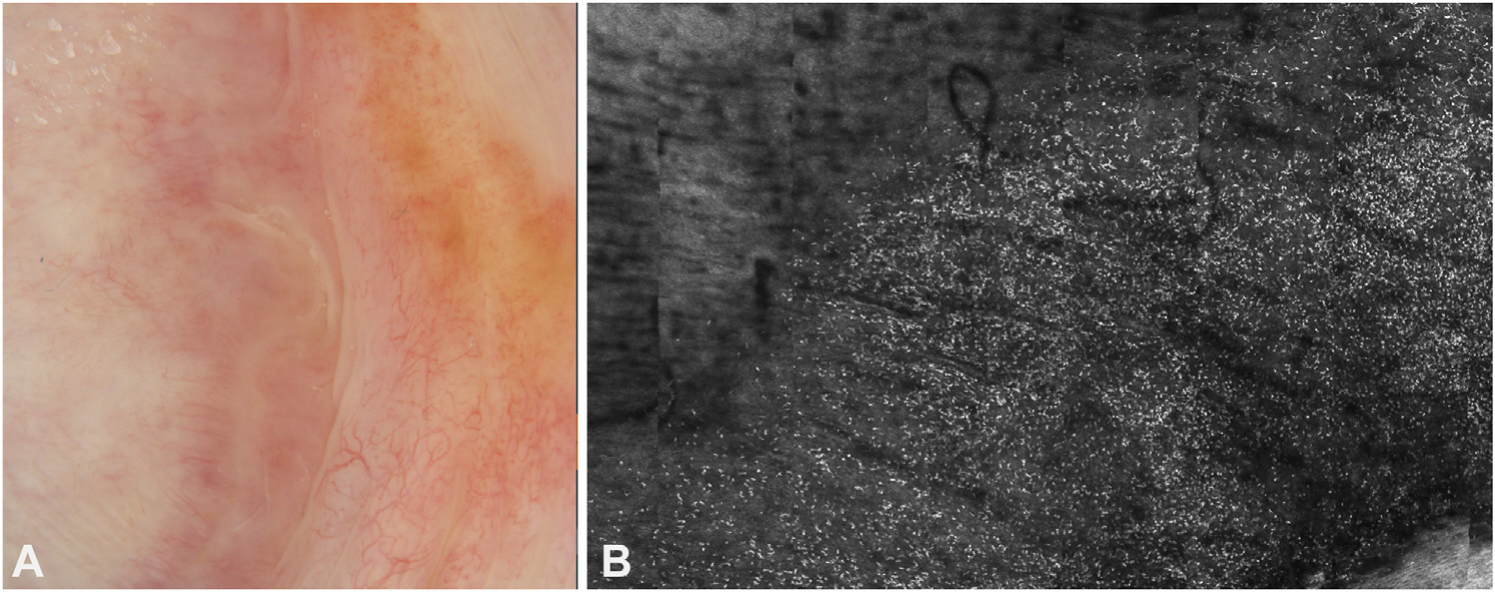
**A,** Dermoscopic pictures before treatment. Note marked erythema and telangiectatic vessels, compared to Fig 2, *A*. **B,** Dermal reflectance confocal microscopy (*RCM*) images of case 1 taken at the onset of monitoring (3 years prior), showing a pronounced dermal inflammatory infiltrate. Notice the small, slightly irregular but uniformed sized hyper-reflective cells within the dermis (inflammatory cells) against a background of fine linear blood vessels. Compare this to Fig 3, *B*, taken 3 years later, following alternating treatments with topical tacrolimus and fluocinonide. (Vivascope 1500, Caliber I.D.; Imaging & Diagnostics).

**Fig 2:**
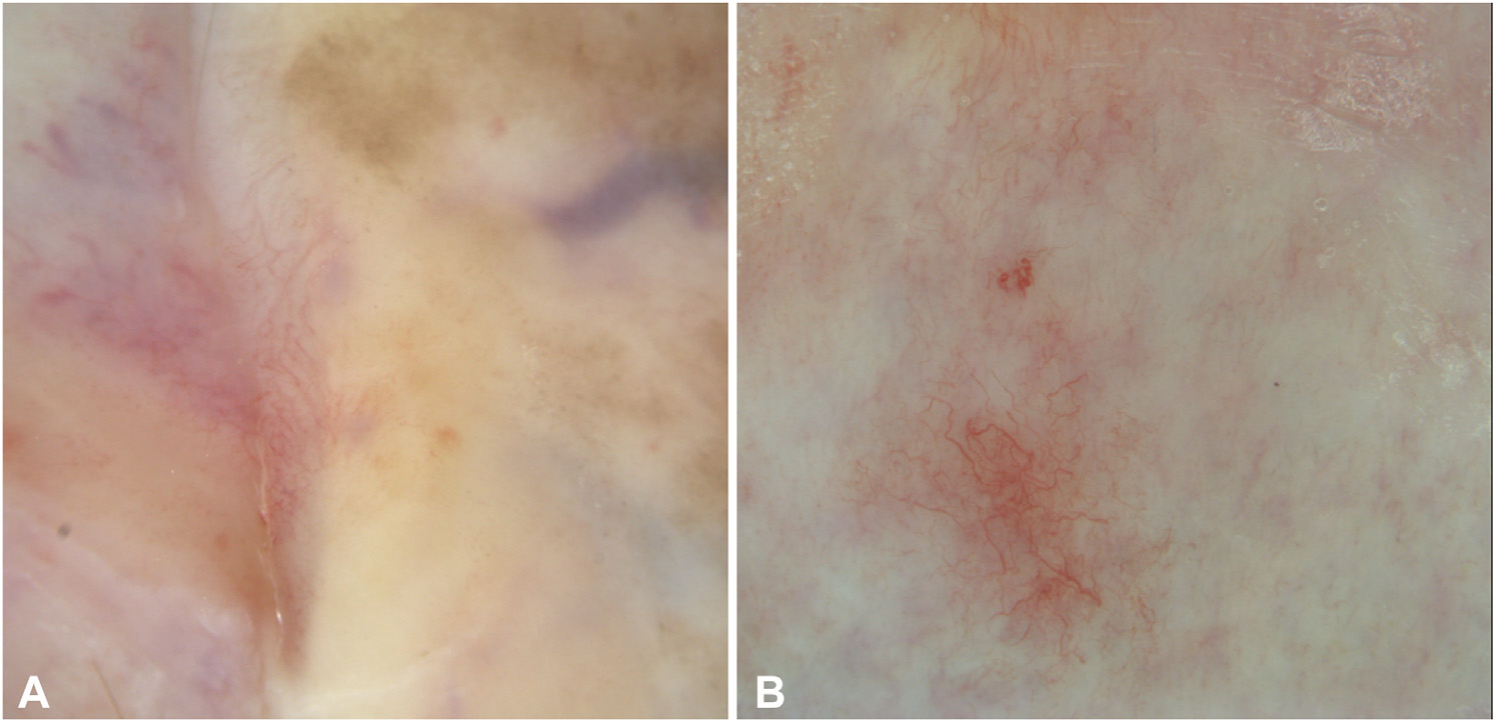
Dermoscopic images of Lichen sclerosus case 1. **A,** Case 1: Prominent telangiectasia with a predominant branching pattern is visible. The background is *white* to *pink* with subtle pigmented granularity. A *red-purple* blotch is present in the *right upper corner*. **B,** Case 2: Similar prominent telangiectasia with a predominant branching pattern is observed. (Vivacam attached to Vivascope 1500, Caliber I.D.; Imaging & Diagnostics).

A 68-year-old man with a history of basal cell carcinoma presented with balanitis and 2 white patches on the dorsal foreskin and head of the penis, clinically consistent with lichen sclerosus (LS). He was treated with fluocinonide 0.05% ointment, tacrolimus 0.1% ointment, and vitamin D supplementation (50,000 IU weekly). His follow-up included total body skin examinations every 6 months and yearly reflectance confocal microscopy (RCM) for the LS lesions. RCM was performed by Vivascope 1500, (Caliber I.D.; Imaging & Diagnostics). After 3 years of yearly monitoring, RCM showed improvement in inflammation (Fig 1) due to treatment and no signs of neoplasia or carcinoma in situ (no honeycomb disarray). The size of the lesions also decreased over time (Fig 2).

### Case 2

**Fig 3:**
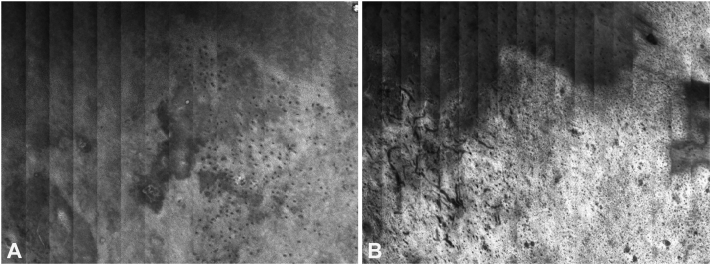
Reflectance confocal microscopy (*RCM*) findings in case 1. **A,** The stratum spinosum is shown, displaying the typical honeycomb pattern. **B,** Deeper RCM sections reveal coarse fibrosis with prominent vascularity, both horizontally and perpendicularly. The pattern observed, particularly in the right half of (**B**), might initially, for those new to RCM, suggest an atypical honeycomb pattern. Thus, carefully reviewing a stack or all RCM mosaics sequentially to orient oneself and confirm the anatomic level is very important. (Vivascope 1500, Caliber I.D.; Imaging & Diagnostics).

An 87-year-old man presented with a pruritic red patch and sclerosis on the head of the penis, clinically diagnosed as balanitis xerotica obliterans. RCM revealed a typical honeycomb pattern (Fig 3) without significant hyperkeratosis and mild disruption of the dermoepidermal junction with a prominent lichenoid inflammatory infiltrate, consistent with inflamed LS, which can show prominent interface activity. Treatment involved clobetasol 0.05%. Subsequent RCM imaging showed a reduction in inflammation and no evidence of neoplastic transformation.

## Confocal and dermoscopic findings

Both cases exhibited characteristic dermoscopic features of LS,^1^ including pronounced telangiectasia and a white to slightly pink background in both instances. Additionally, case 1 displayed focal red to purple blotches, likely indicative of subcutaneous hemorrhage, possibly related to pruritus. There was also subtle pigmented granularity noted. The vascular pattern was predominantly branching, with localized areas exhibiting looped blood vessels and a few dot vessels. RCM findings were consistent in both cases, revealing a normal, regular honeycomb pattern in the epidermis, which appeared thinned, along with inflammation at the dermoepidermal junction, dermal fibrosis, and deep dermal inflammatory infiltrates. Notably, to an untrained observer, the dense dermal fibrosis coupled with prominent blood vessels could be mistaken for an irregular honeycomb pattern with dermal papillae, potentially leading to an erroneous diagnosis of squamous cell carcinoma in situ, if the absence of nuclear structures is not carefully noted.

## Key message

LS is a chronic condition affecting the anogenital area, marked by white patches and a risk of turning into squamous cell carcinoma in 1% to 6% of cases. Regular monitoring is vital due to this risk.^2^^,^^3^ Biopsies are commonly used for diagnosis but can be problematic in sensitive areas.^3^ RCM is highlighted as a noninvasive tool for assessing and monitoring LS. It can look deeper into the skin and measure inflammation and skin thickness. Studies have shown RCM can identify specific features of LS, aligning with histological characteristics.^4^^,^^5^ RCM's ability to monitor therapy and detect precancerous changes in delicate regions highlights its importance in clinical practice. Dermatologists and confocal microscopists would benefit from familiarity with RCM findings in LS but should also be aware of some pitfalls that may arise when attempting to rule out neoplasia, including the unusual appearance of thick dermal collagen and increased vascularity that can be mistaken for a honeycomb disarray with dermal papillary openings and consequently a misdiagnosis of squamous cell carcinoma in situ.

## Conflicts of interest

None disclosed.
